# Gestational PBDE concentrations and functional connectivity in adolescents: The HOME Study^☆^

**DOI:** 10.1016/j.ijheh.2026.114745

**Published:** 2026-01-15

**Authors:** Jonathan Dudley, Kimberly Yolton, Alex D. Edmondson, Yingying Xu, Aimin Chen, Jeffrey R. Strawn, Joseph M. Braun, Andreas Sjodin, Bruce P. Lanphear, Kim M. Cecil

**Affiliations:** a Imaging Research Center, Cincinnati Children’s Hospital Medical Center, Cincinnati, OH, USA; b Department of Pediatrics, Cincinnati Children’s Hospital Medical Center, University of Cincinnati College of Medicine, Cincinnati, OH, USA; c Department of Radiology, Cincinnati Children’s Hospital Medical Center, University of Cincinnati College of Medicine, Cincinnati, OH, USA; d Department of Environmental and Public Health Sciences, University of Cincinnati College of Medicine, Cincinnati, OH, USA; e Department of Biostatistics, Epidemiology and Informatics, University of Pennsylvania Perelman School of Medicine, Philadelphia, PA, USA; f Department of Psychiatry and Behavioral Neuroscience, University of Cincinnati College of Medicine, Cincinnati, OH, USA; g Department of Epidemiology, Brown University School of Public Health, Providence, RI, USA; h Division of Laboratory Sciences, National Center for Environmental Health, Centers for Disease Control and Prevention, Atlanta, GA, USA; i Department of Health Sciences, Simon Fraser University, Burnaby, BC, Canada

**Keywords:** Polybrominated diphenyl ether flame, retardants, Gestational, Adolescent, Visual cortex, Functional magnetic resonance imaging, Functional connectivity, Mediation

## Abstract

**Background:**

Gestational polybrominated diphenyl ethers (PBDE) exposure is linked with adverse neurobehavioral outcomes in children, but scientists do not fully understand how these flame retardants damage the developing brain.

**Objective:**

We estimated the association of gestational PBDE serum concentrations and intrinsic functional connectivity in 143 adolescents from the Health Outcomes and Measures of the Environment (HOME) Study, a prospective pregnancy and birth cohort enrolling from 2003 to 2006.

**Methods:**

We measured maternal serum concentrations of five PBDE congeners and created a summary variable (Σ_5_BDE) during pregnancy. At age 12 years, we acquired high resolution anatomical and resting state functional magnetic resonance neuroimaging to examine the relationship between gestational concentrations and intrinsic functional connectivity, adjusting for covariates. We assessed behaviors, mental health symptoms, and executive function using self- and caregiver-reports. We examined whether functional connectivity patterns mediated the observed associations between gestational PBDE concentrations and neurobehavioral outcomes.

**Results:**

Higher gestational BDE-153 serum concentrations were associated with higher local correlation in the left inferior lateral occipital cortex. Higher gestational PBDE serum concentrations (BDE-28, −47, −99, −100, and the Σ_5_BDE) were associated with reduced global correlation in the primary visual cortex. Seed-to-voxel connectivity patterns showed significant mediation for associations between Σ_5_BDE and neurobehavioral outcomes.

**Discussion:**

Higher gestational PBDE serum concentrations were associated with diminished connectivity in the primary visual cortex. Connectivity patterns also mediated the relationship between gestational Σ_5_BDE concentrations and neurobehavioral outcomes at age 12. These findings suggest that gestational PBDE exposure alters how the brain processes visual information across global networks.

## 1. Introduction

Polybrominated diphenyl ethers (PBDEs) were employed as additive flame retardants for consumer and commercial products. Despite a voluntary phase-out of PBDE production circa 2006–2013, products containing PBDEs remain ubiquitous as dust particles are released from these products and absorbed into the body (Johnson et al. 2010, 2013). PBDE congeners, which bioaccumulate in fatty tissues in humans, have half-lives from 1 to 7 years (Costa et al., 2014; Geyer et al., 2002; Herbstman and Mall, 2014; Sjodin et al., 2020; Trudel et al., 2011). While exposures declined by a factor of 2–3 since the phase-out, a recent NHANES analysis found longer population half-lives suggesting that Americans still experience substantial ongoing PBDE exposures (Sjodin et al., 2024).

PBDE exposure during early brain development has been associated with cognitive deficits including language development (Ding et al., 2015), intelligence (Vuong et al., 2017b), reading ability (Zhang et al., 2017), working memory (Sagiv et al., 2015), attention and executive function (Sagiv et al., 2015; Vuong et al., 2016) in children. Studies have linked PBDE exposures with childhood attention deficit hyperactivity disorder (ADHD) symptoms that persist into adolescence (Braun et al., 2017b; Cecil et al., 2024; Chen et al., 2014; Cowell et al., 2015; Sagiv et al., 2015; Zhang et al., 2017). Gestational PBDE exposure also increases the risk of developing depressive and anxiety symptoms, including panic and social anxiety (Strawn et al., 2022).

*In vitro* and animal models demonstrate evidence of direct and indirect mechanisms of neurotoxicity and long-lasting behavioral alterations originating from *in utero* PBDE exposures (Costa et al., 2014; Herbstman and Mall, 2014; Vuong et al., 2018b). Concerns regarding adverse neurobehavioral outcomes associated with PBDE exposures have prompted novel lines of investigation to better understand the biological pathways underlying the neurotoxic effects of PBDEs.

Non-invasive, magnetic resonance imaging (MRI)-based neuroimaging methods such as resting state functional magnetic resonance imaging (rsfMRI), provide network-level insight into brain function and organization. This technique measures low-frequency oscillations in the blood oxygen-level dependent (BOLD) signal that are strongly associated with spontaneous neural activity due to neurovascular coupling (Lv et al., 2018). In rsfMRI, participants lie within the scanner without performing a specific task. BOLD signal changes are recorded from each image voxel (i.e., a three-dimensional pixel) and the temporal correlation of neural activity (termed “connectivity”) between different regions of the brain are quantified. By determining the degree of local and global connectivity in the brain, inferences about network functioning can be constructed. We estimate the association of gestational PBDE serum concentrations and intrinsic functional connectivity in adolescents enrolled in the Health Outcomes and Measures of the Environment (HOME) Study. To understand the significance, we explore whether functional connectivity patterns mediate the established associations of gestational PBDE serum concentrations and neurobehavioral outcomes.

## 2. Materials and methods

### 2.1. Study participants

**Figure 1:**
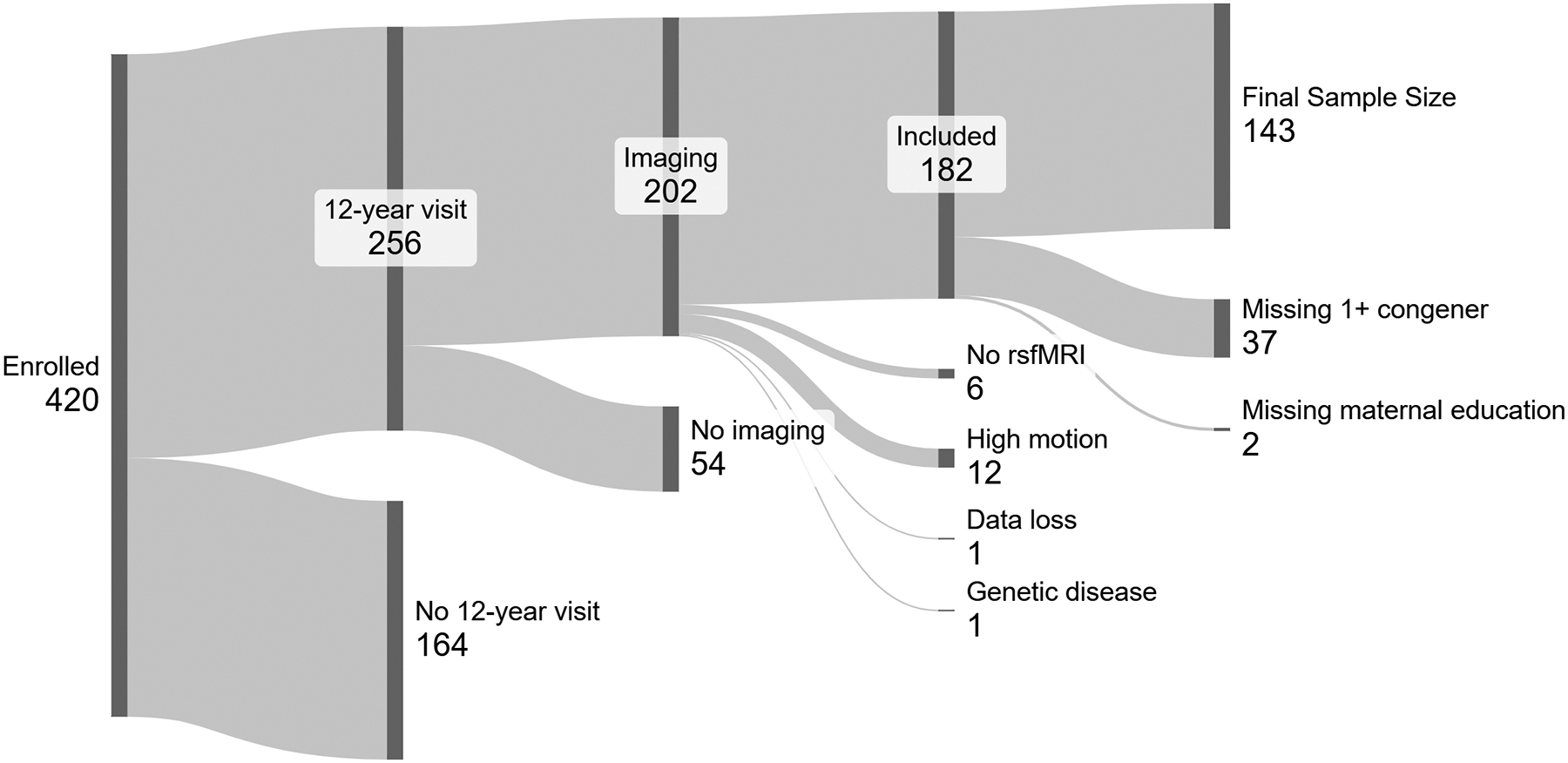
Flow diagram illustrating missing data and selection of participants for analysis of gestational Σ_5_BDE concentration and resting state functional MRI (rsfMRI).

The HOME Study, a prospective pregnancy and birth cohort within the metropolitan area of Cincinnati, Ohio, USA, enrolled pregnant women (N = 468) at 16 ± 3 weeks of gestation between 2003 and 2006. Eligible women were over 18 years, no more than 19 weeks’ gestation, not taking medications for seizure or thyroid disorders, negative HIV status, and living in a house built before 1978 to assess lead hazards. Additional details about the study enrollment criteria, exposure measurements, and neurobehavioral assessments are published (Braun et al. 2017a, 2020). A total of 420 children (11 sets of twins included) completed at least one subsequent study visit from birth to age 12 years. A total of 256 caregiver-adolescent pairs completed the study visit at age 12 years with 202 participating in the imaging examination, but only 196 adolescents completing the rsfMRI sequence. The rsfMRI datasets were excluded for excessive motion (>0.4 mm mean motion *and/or* >40 % of frames censored) (n = 12) and storage archive corruption (n = 1). One participant with useable fMRI data was excluded due to a known genetic disease. Missing serum concentrations for BDE-28, BDE-47, BDE-99, BDE-100, BDE-153 resulted in 37, 9, 10, 11, and 9 participants being excluded from analyses, respectively. The large number missing for BDE-28 occurred from inadequate samples available for analyses. Missing reported maternal education at baseline excluded 2 participants from the analyses resulting in 143 adolescents with complete rsfMRI data, PBDE concentrations and demographic covariates. The attrition of participants and their data is illustrated (Fig. 1). All data collection was completed prior to the COVID-19 pandemic.

### 2.2. Ethical considerations

The institutional review boards at Cincinnati Children’s Hospital Medical Center (CCHMC) and the enrolling delivery hospitals approved this study. The Centers for Disease Control and Prevention (CDC) laboratory’s involvement did not constitute engagement in human-subjects research. Caregivers provided written informed consent for their own participation as well as their child. At age 12 years, adolescents provided informed assent.

### 2.3. PBDE quantification from maternal serum

We collected whole blood samples from pregnant women at 16 weeks or 26 weeks gestation. Sera were separated and stored at −80 °C until time for measurement of BDE congeners (−28, −47, −99, −100 and −153) using gas chromatography/isotope dilution high-resolution mass spectrometry (Jones et al., 2012; Sjodin et al., 2004; Sjodin and Pirkle). Details about the PBDE measurements, including quality control/assurance procedures, and lipid normalization, have been previously described (Vuong et al., 2017a). PBDE concentrations below the limit of detection (LOD) were substituted with LOD/√2 (Hornung and Reed, 1990). Serum PBDE concentrations were standardized by serum total lipid concentrations in accounting for the lipophilic nature of these chemicals (O’Brien et al., 2016; Phillips et al., 1989). A summary exposure measure (Σ_5_BDE) was generated as the arithmetic sum of five PBDE congeners (BDE-28, −47, −99, −100 and −153) concentrations, an approach that has been used in previous investigations of PBDEs (Cecil et al. 2024, 2025; Hartley et al., 2022; Strawn et al., 2022; Vuong et al., 2018a).

### 2.4. Neurobehavioral assessments

We assessed several domains of adolescent neurobehavior at the age 12 study visit. Briefly, we assessed adolescent executive function obtaining adolescent and caregiver reports using the Behavior Rating Inventory of Executive Function, second edition (BRIEF-2)(Gioia et al., 2015), problem and adaptive behaviors using the Behavioral Assessment System for Children, third edition (BASC-3)(Reynolds and Kamphaus, 2015), and social behaviors using the Social Skills Improvement System (SSIS) scale (Gresham et al., 2008). We assessed adolescent social behaviors by administering to the caregivers the Social Responsiveness Scale (SRS-2) (Constantino and Gruber, 2012; Constantino and Todd, 2005) We administered to the adolescent the Spence Children’s Anxiety Scale (SCAS) (Spence, 1998), Screen for Child Anxiety Related Disorders (SCARED) (Birmaher et al., 1997), the Child and Adolescent Memory Profile (ChAMP) (Sherman and Brooks, 2015), and Grooved Pegboard (Instrument, 2014) to assess anxiety, memory and fine motor skills, respectively. Adolescents self-reported depressive symptoms with the Children’s Depression Inventory-II (CDI-2) (Kovacs, 1992). The administration of several of these instruments (BRIEF-2, BASC-3, SSiS, SCARED) has been previously reported in the HOME Study for the age 12 study visit (Cecil et al. 2024, 2025; Hartley et al., 2022; Strawn et al., 2022). The SCAS (Spence, 1998) assesses six domains of anxiety including separation anxiety, obsessive compulsive disorder, social phobia, panic/agoraphobia, physical injury fears and generalized anxiety, as well as a total anxiety score. The SCAS contains 44 items measuring self-reported symptoms of anxiety, each scoring between 0 (never) and 3 (always) points. Points are summed to produce a score for each of the six subscales and a Total Anxiety score. Scoring is converted to age- and gender-standardized T-scores with mean values of 50 and standard deviation values of 10. The SRS-2 is a 65-item questionnaire that assesses behavioral and social-communicative traits used to identify the presence and severity of social impairment for both the general (non-clinical) population and in clinical settings (Bolte et al., 2008; Constantino and Todd, 2005; Frazier et al., 2014). SRS raw scores were created utilizing publisher-supplied software. Sex-specific T-scores with a mean of 50 and standard deviation of 10 were determined based upon the publisher’s instructions with higher scores representing more severe impairment.

### 2.5. Imaging acquisition

We acquired neuroimaging data for this study at Cincinnati Children’s Hospital Medical Center using a Philips Ingenia 3 T field strength MR System (Philips Healthcare, Best, The Netherlands) equipped with a 32-channel head coil. Anatomical MRI images of the brain were acquired using a three-dimensional T1-weighted fast Fourier echo sequence with a repetition time (TR) of 8 ms, echo time (TE) of 3.7 ms, flip angle of 8°, field of view dimensions of 256 mm by 224 mm by 192 mm, voxel size of 1 mm by 1 mm by 1 mm, 192 slices, sagittal slice orientation, a sensitivity encoding (SENSE) factor 2 (S reduction) applied and the sequence duration lasting 6 min, 20 s. We acquired two consecutive rsfMRI sequence runs using a two-dimensional T2*-weighted, single-shot, echo planar imaging sequence with a TR of 2000 ms, TE of 35 ms, flip angle of 90°, field of view dimensions of 256 mm by 256 mm, acquisition matrix of 64 by 62, reconstruction matrix 64 × 64, in-plane resolution of 4 mm by 4, slice thickness of 5 mm, 35 slices, 165 dynamics, spectral presaturation with inversion recovery (SPIR) fat suppression applied with an offset of 135 Hz, slice orientation transverse (parallel to the orbitofrontal cortex line), SENSE factor of 2 (P reduction) was applied and the sequence duration lasting 5 min and 36 s (11:12 in total for the two runs). Participants were instructed to rest supine in the scanner without motion and gaze at a white plus sign (+) on a black background for the duration of each rsfMRI sequence run.

### 2.6. Image post-processing

We performed image processing and analyses using the functional connectivity software toolbox for correlated and anti-correlated brain networks referred to as CONN (RRID:SCR_009550) release 22.a (Chai et al., 2012; Nieto-Castanon and Whitfield-Gabrieli, 2021; Whitfield-Gabrieli and Nieto-Castanon, 2012) and Statistical Parameter Mapping software version 12 (SPM12) (Penny et al., 2007) (RRID: SCR_007037) release 12.7771. Functional data were realigned using SPM12’s *realign & unwarp* procedure (Andersson et al., 2001), where all frames were rigidly co-registered to the first frame of the first session (Friston et al., 1995), and resampled using b-spline interpolation to correct for motion and magnetic susceptibility interactions. Frames with framewise displacement above 0.9 mm or global BOLD signal changes above 5 standard deviations were demarcated as outliers using Artifact Detection Tools (RRID:SCR_005994) within the CONN toolbox (Nieto-Castanon, 2022; Power et al., 2014; Whitfield-Gabrieli et al., 2011). We normalized functional and anatomical data into standard Montreal Neurological Institute (MNI) template space, segmented into grey matter, white matter, and cerebrospinal fluid (CSF) tissue classes, and resampled to 2 mm isotropic voxels following a direct normalization procedure (Calhoun et al., 2017; Nieto-Castanon, 2022) using SPM unified segmentation and normalization algorithm (Ashburner, 2007; Ashburner and Friston, 2005) with the default IXI-549 tissue probability map template. Functional data were spatially smoothed with a Gaussian kernel of 6 mm full width half maximum (FWHM). The following signals were regressed from the functional data: 5 CompCor (Behzadi et al., 2007; Chai et al., 2012) components each from white matter and CSF compartments, 6 framewise motion parameters and their first order derivatives, outlier scans, and a hemodynamic response convolved task timing (constant “effect” of the resting state condition) and its first order derivatives. Data were then linearly detrended and bandpass filtered between 0.008 Hz and 0.09 Hz. Participant datasets (n = 12) were excluded for excessive motion (>0.4 mm mean motion *and/or* >40 % of frames censored). No significant associations were observed between functional connectivity and mean motion or frames censored after exclusion and denoising (Ciric et al., 2018).

Voxelwise connectivity analyses allow researchers to test hypotheses about functional networks by creating “maps” of summary measures of connectivity for each voxel. Because the effects of gestational PBDE concentrations on brain function and organization in adolescence are not well characterized, we chose a voxelwise approach (as opposed to an *a priori* seed based approach) in this study and report two broad measures of network organization: local correlation (LCOR) and global correlation (GCOR). As the names imply, LCOR maps measure how strongly each voxel is connected to other nearby voxels while GCOR maps measure the average connectivity strength of each voxel to all other voxels in the brain. Significant clusters from these voxelwise analyses can then be used in seed-based *post hoc* analyses to further investigate the network connectivity patterns. LCOR maps were estimated as the weighted average of all short-range connections between a given voxel and a 25 mm FWHM Gaussian neighborhood area (Deshpande et al., 2009). GCOR maps were estimated as the average of all short- and long-range connections between a given voxel and the rest of the brain (Saad et al., 2013).

### 2.7. Statistical analyses

#### 2.7.1. Descriptive statistics

Descriptive statistics were used to summarize and examine the data distribution of household, maternal, and adolescent characteristics and identify potential outliers across exposure measures. Means and standard deviations or medians and interquartile ranges or geometric means and their 95 % confidence intervals are reported for the continuous variables as appropriate; frequencies and percentages are reported for categorical variables. We log_2_-transformed the lipid-normalized PBDE concentrations before further statistical analyses to reduce the influence of extreme values, therefore the regression coefficients correspond to each doubling of the PBDE concentration.

#### 2.7.2. Neurobehavioral correlates of gestational PBDE concentrations

For each of the gestational PBDE serum concentrations (log_2_BDE-28, −47, −99, −100, −153, or log_2_Σ_5_BDE), we sought to examine univariate associations with 24 key neurobehavioral outcome composite indexes providing T-scores obtained from the BASC-3, BRIEF-2, SCARED, SSiS, SRS, SCAS and CDI-2. Associations were assessed using bivariate Pearson correlation.

#### 2.7.3. Neural correlates of gestational PBDE concentrations

Group-level analyses of functional connectivity metrics were carried out in CONN to test associations between gestational PBDE serum concentration and voxelwise connectivity metrics (LCOR or GCOR). A separate general linear model was fit for each voxel with the connectivity metric as the dependent variable, one of the gestational PBDE concentration values (log_2_BDE-28, −47, −99, −100, −153, or log_2_Σ_5_BDE) as the independent variable of interest, and age, sex and maternal education at baseline as additional explanatory variables selected *a priori* based on prior literature. Age and education serve as potential confounders and child sex as a precision variable. We considered other covariates, including pre-pregnancy body mass index, ethnicity, occupation, income, and primipara but they did not act as confounder or were highly correlated with maternal education and thus not adjusted for in the models. Cluster-level inferences were based on parametric statistics from Gaussian Random Field theory; results were thresholded using a combination of a cluster-forming p < 0.001 voxel-level threshold and a false discovery rate (FDR): p-FDR <0.05 cluster-size threshold.

#### 2.7.4. Post-hoc mediation analyses

Lastly, we sought to examine whether functional connectivity characteristics mediated the observed associations between log_2_Σ_5_PBDE) and neurobehavioral outcome T-scores. For these analyses, 16 participants were excluded because their imaging session was conducted more than three months after their 12-year neurobehavioral testing (these participants had orthodontics at their initial 12-year visit and were rescheduled for imaging after removal). Mediation analyses were performed using the CANLAB Multilevel Mediation and Moderation (M3) Toolbox (https://github.com/canlab/MediationToolbox/). The mediation effect (also known as the indirect effect) is termed *ab* and was computed as the product of “path *a*” – the coefficient for the effect of PBDE concentration on the mediator – and “path *b*” – the coefficient for the effect of the mediator on the neurobehavioral outcome score controlling for the effects of the exposure variable. This is mathematically equivalent to the total effect minus the direct effect (*c* - *c’*: the change in coefficient for the effect of PBDE concentration on the outcome score when the effect of the mediator is controlled for in the analysis).

For log_2_BDE153 – which had a significant effect on the *local* correlation in the left inferior lateral occipital cortex (see Results section 3.4 Neural Correlates of Gestational PBDE concentrations) – we hypothesized that the average connectivity strength *within* the significant cluster would exhibit a mediating effect on exposure-outcome associations. Five mediation models were run; in each model the independent variable was log_2_BDE153, the mediator variable was the average connectivity within the aforementioned cluster, and the dependent variable was one of the five outcome measures found to have significant associations with log_2_BDE153 concentration (see Table 3). Significance of the indirect effect *ab* was determined via bootstrapping with 5000 bootstrap samples upon replacement (Shrout and Bolger, 2002).

For log_2_Σ_5_BDE – which had a significant effect on the *global* correlation strength of voxels in a cluster in the left primary visual cortex (see Results section 3.4 Neural Correlates of Gestational PBDE concentrations), meaning higher concentrations were associated with (on average) lower connectivity between this region and the rest of the brain. Accordingly, we hypothesized that *patterns* of connectivity associated with this cluster would mediate the observed relationships between log_2_Σ_5_BDE concentrations and neurobehavioral outcomes. To test this hypothesis, we employed a *multivariate* mediation analysis framework (Chen et al., 2018; Geuter et al., 2020). The M3 toolbox was used to construct principal directions of mediation (PDM) – orthogonal components of a high-dimensional intermediary variable that can be conceptualized as connectivity patterns. We ran a multivariate mediation model for each significant association observed between neurobehavioral outcomes and gestational exposure to log_2_Σ_5_BDE (15 models in total).

For each model, the independent variable was gestational log_2_Σ_5_BDE concentration, the dependent variable was the given neurobehavioral outcome score, and the high-dimensional intermediary variable was a seed-to-voxel connectivity map based on the results from the voxelwise analyses described above in section 2.7.3. Specifically, the average residual BOLD signal within the cluster showing significant associations between log_2_Σ_5_BDE and GCOR (see section 3.4 Neural correlates of Gestational PBDE concentrations) was taken as the seed and the connectivity strength was computed for every voxel in the brain grey matter (N = 158,156) for each participant. The dimensionality of this variable was reduced using singular value decomposition to a number of components that explained 90 % of the variance in the data; 15 PDMs were then constructed for each exposure-outcome pair and the mediation effect calculated.

We determined the statistical significance of the multivariate mediation model was determined by permutation testing via participant-wise shuffling of the seed-to-voxel connectivity data and subsequent construction of “null” PDMs and their respective indirect effects. Because meaningful indirect effects can be either positive (e.g., a maladaptive response) or negative (e.g., a neurocompensatory mechanism), we compare the sum of the *absolute values* of the indirect effects of all PDMs in the model to those in the permutations. The p-value of the multivariate mediation model is taken as the proportion of the permutations with greater sum of absolute indirect effects across PDMs than the real case; 1000 such permutations were performed. Voxelwise weights corresponding to each PDM were obtained and their statistical significance determined via bootstrapping with 5000 bootstrap samples upon replacement.

## 3. Results

### 3.1. Descriptive statistics – participants

For the 143 adolescents with complete rsfMRI data, PBDE concentrations and demographic covariates, the average age was 12 years (12.3 ± 0.7) at the time of the study visit, 58.7 % were female; 46.9 % had mothers who had attained a bachelor’s degree or higher at baseline, which increased to 52.5 % at the adolescent study visit (Table 1). For comparison of maternal demographics, we summarized variables from the 238 adolescents not included in the rsfMRI imaging subcohort. These result from 164 who did not participate in the age 12 study visit, 54 who participated in the age 12 study visit but not the imaging examination, and the 20 who participated in the imaging examination but were excluded for motion, data loss, no rsfMRI or genetic disease (Fig. 1). The 143 participants included in the Σ_5_BDE and BDE-28 analyses were more likely to be female, and non-Hispanic Black from those excluded.

### 3.2. Gestational PBDE concentrations

We detected five PBDE congeners in at least 88.8 % of the maternal serum samples. The geometric means of PBDE serum concentrations were 1.1, 22.0, 4.9, 4.0, 4.9 and 38.1 ng/g lipid for BDE-28, −47, −99, −100, −153 and Σ_5_, respectively (Table 2). The 5 congeners were moderately to strongly correlated, with Pearson’s correlation coefficients ranging from 0.46 (between BDE-99 and BDE-153) to 0.93 (between BDE-47 and BDE-99). As expected, they were also strongly correlated with Σ_5_BDE with Pearson’s correlation coefficients ranging from 0.73 to 0.97.

### 3.3. Associations of gestational PBDE concentrations with neurobehavioral outcomes

Table 3 shows the results from univariate analyses of associations between gestational PBDE serum concentrations and neurobehavioral outcomes. Log_2_BDE-28 tended to have the strongest associations with neurobehavioral outcomes, followed by log_2_Σ_5_BDE while associations tended to be weakest for log_2_BDE-100 and log_2_BDE-153. Exposure-outcome associations also tended to be stronger for adolescent-rated outcome measures compared to caregiver-rated outcome measures as previously noted in published analyses of the larger cohort (Cecil et al. 2024, 2025; Strawn et al., 2022).

### 3.4. Neural correlates of gestational PBDE concentrations

**Figure 2:**
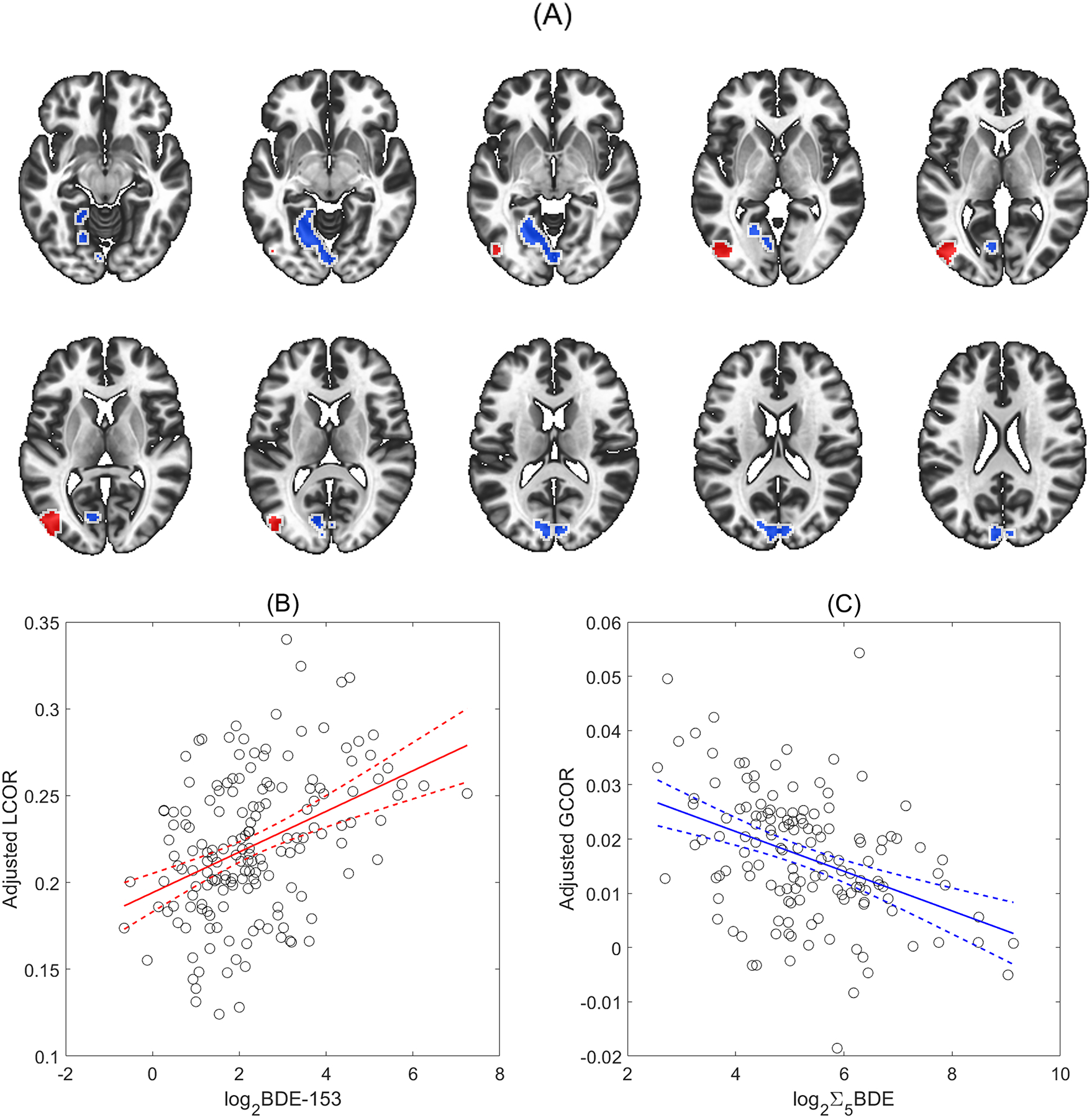
A) Clusters exhibiting significant positive association between local correlation and gestational log_2_BDE-153 (red) and negative associations between global correlation and gestational log_2_Σ_5_BDE (blue). B) Gestational log_2_BDE-153 association with the average local correlation in the corresponding cluster, adjusted for effects of age, sex, and maternal education at baseline. C) Gestational log_2_Σ_5_BDE association with the average global correlation in the cluster, adjusted for effects of age, sex, and maternal education at baseline. Solid and dashed lines in (B) and (C) are the ordinary least squares fit regression line and 95% confidence intervals.

Higher gestational BDE-153 serum concentration was associated with higher local correlation in a single cluster spanning 15 % of the left inferior lateral occipital cortex (Fig. 2A shown in red). The cluster size was statistically significant (p-FDR = 0.003) at 2.50 cm^3^ in volume and centered on the MNI coordinates x = −46, y = −76, z = +6. On average, a doubling of gestational PBDE-153 serum concentration corresponded to 5.3 % higher LCOR in this cluster (95 % confidence interval: 3.4 %–7.2 %), after controlling for age, sex, and maternal education (Fig. 2B).

Greater gestational Σ_5_BDE concentrations were associated with lower GCOR in a single cluster in the occipital lobe (Fig. 2A shown in blue). On average, a doubling of gestational Σ_5_BDE corresponded to 21.9 % lower GCOR in this cluster (95 % confidence interval: 13.5 %–30.3 % reduction), after controlling for age, sex, and maternal education (Fig. 2C). Associations between GCOR and individual PBDE congeners yielded similar results for BDE-28, −47, −99, and −100. For each congener a single cluster in the occipital lobe was identified; the cluster sizes were significant at p-FDR <0.05 for BDE-47 and BDE-100, but not for BDE-28 and BDE-99. Table 4 lists the anatomical structures with which each cluster overlaps. We found no association between GCOR and BDE-153 (cluster p-FDR >0.3).

### 3.5. Post-hoc mediation analyses

For BDE-153 exposure, five mediation analyses were performed – one for each of the exposure-outcome associations previously found as significant (see Table 3). These mediation analyses assessed whether the average connectivity within the significant LCOR cluster (see Fig. 2A in red) exerted a significant indirect effect on the log_2_BDE-153-outcome associations in Table 3. None of the five mediation analyses were found to have significant indirect effects.

For Σ_5_BDE, fifteen *multivariate* mediation analyses were performed – one for each of the exposure-outcome associations previously found as significant (see Table 3). These multivariate mediation analyses assessed whether patterns of connectivity between the significant GCOR cluster (see Fig. 2A in blue) and the rest of the brain exerted a significant indirect effect on the log_2_Σ_5_BDE-outcome associations in Table 3.

**Figure 3:**
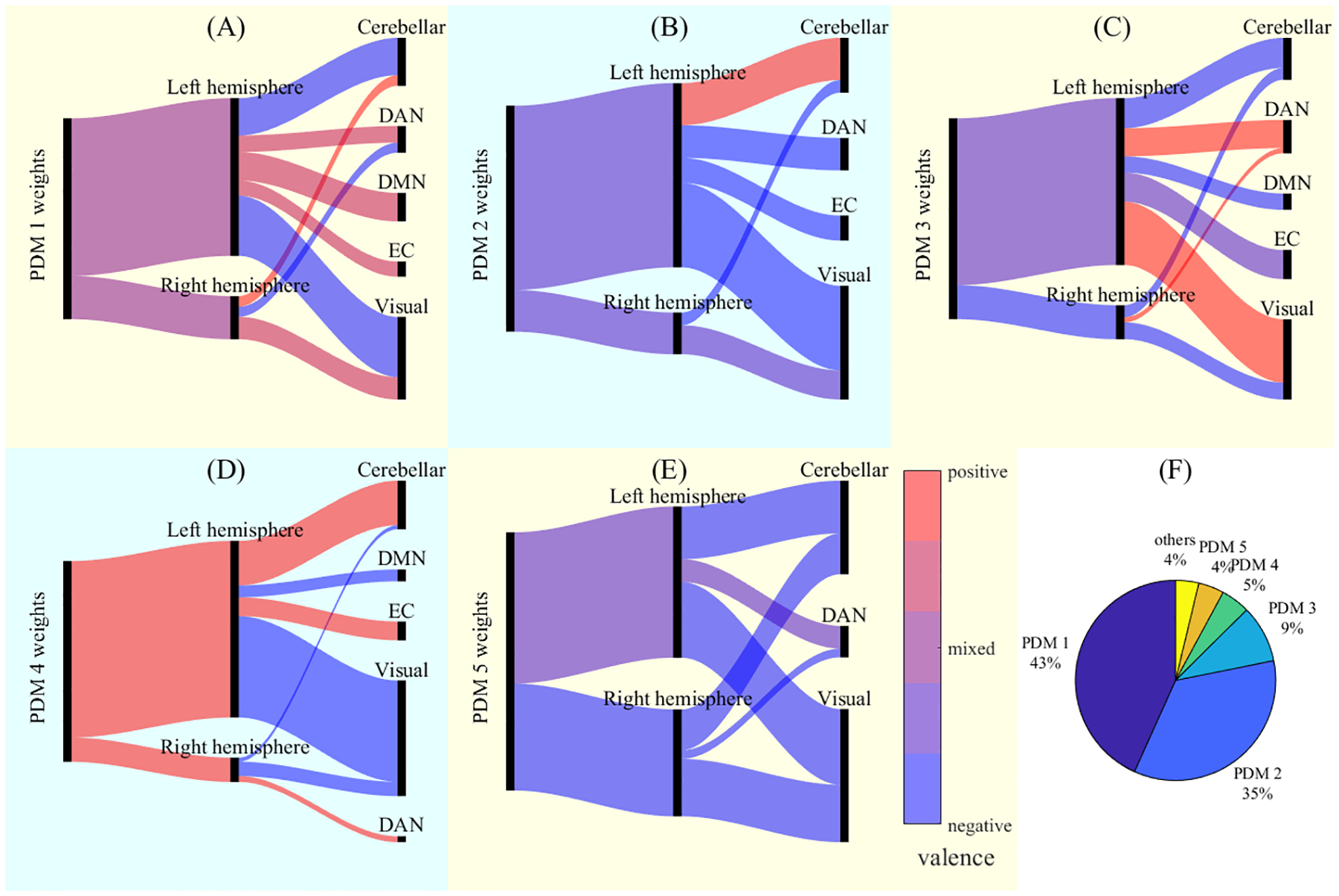
A multivariate mediation model showing a significant indirect effect on the observed positive association between gestational Σ_5_BDE concentration and Social Responsiveness Scale Total T-score obtained at the age 12 study visit. Parts A-E show the distribution of significant weights across hemispheres and functional networks for each of the first five PDMs. Heights of the vertical black bars are proportional to the volume of significant weights. Links are color coded from blue to purple to red and indicate the valence of the weights; positive/negative weights indicate that higher levels of gestational Σ_5_BDE exposure are associated with increased/decreased connectivity between the primary visual cluster (Figure 1A) and the given region. Yellow backgrounds indicate the PDM has a positive path b and thus explain some of the positive association between gestational Σ_5_BDE concentration and SRS total T-score; blue backgrounds indicate the PDM has a negative path b and thus exert an ameliorating effect. Part (F) shows the percentage of the model’s indirect effect explained by each PDM. Functional network abbreviations: DAN – dorsal attention network, DMN – default mode network, EC – executive control network.

Seven of these multivariate mediation models were found to have significant indirect effects for the following outcome measures: BASC-BSI-caregiver reported (p = 0.009), SCARED total (p = 0.007), SCAS total (p = 0.008), CDI total (p = 0.002), SRS total (p = 0.001), BRIEF-CRI (p = 0.016), and BRIEF-GEC (p = 0.046). Table 5 summarizes the results of these mediation models as well as the characteristics of the first five PDMs for each model. Fig. 3A–E depicts the distribution of significant voxel weights for the first five individual PDMs of the gestational Σ_5_BDE concentration-SRS total T-score mediation model. Heights of the vertical black bars are proportional to the volume of the significant weights present in the given hemisphere/network. The links are color coded to represent the direction of significant weights with red corresponding to mostly/entirely positive weights, blue corresponding to mostly/entirely negative weights, and shades of purple indicating a mixture of positive and negative weights.

Fig. 3A indicates that higher Σ_5_BDE concentration was broadly associated with both increased and decreased connectivity (i.e., mixed valence) that was largely left lateralized; more specifically, higher Σ_5_BDE concentration was associated with lower connectivity between the GCOR cluster and regions in left visual and cerebellar regions and right dorsal attention network regions. Higher Σ_5_BDE concentration was simultaneously associated with higher connectivity in right visual and cerebellar regions and left dorsal attention network, default mode network, and executive control regions. The positive path b (as indicated by the yellow background) means that this pattern of connectivity was associated with higher SRS scores when controlling for Σ_5_BDE concentration and could indicate a maladaptive response.

Conversely, Fig. 3B shows a PDM with a negative path b (blue background), meaning the pattern of connectivity was associated with lower SRS scores when controlling for Σ_5_BDE concentration, potentially indicating a neurocompensatory response. For this PDM, higher Σ_5_BDE concentration was associated lower connectivity of the GCOR cluster overall; specifically, higher Σ_5_BDE concentration was associated with lower connectivity between the GCOR cluster and regions in bilateral visual, left dorsal attention network, left executive control, and right cerebellar regions as well as with higher connectivity between the GCOR cluster and left cerebellar regions. Subsequent PDMs can be interpreted similarly, but their relative importance diminishes rapidly, as seen in figure Fig. 3F which shows the percentage of the overall model’s indirect effect explained by each PDM. Figures for other Σ_5_BDE multivariate mediation models are provided in the supplemental material (Supplemental Fig. 1–6).

## 4. Discussion

We employed rsfMRI to indirectly measure spontaneous neural activity and determine if gestational PBDE concentration alters brain network functioning. Our voxelwise approach for assessing intrinsic functional connectivity was exploratory and data driven. We found higher gestational BDE-153 concentrations were associated with higher local correlation in a single cluster of voxels located in the left inferior lateral occipital cortex. In this cluster, a doubling of gestational BDE-153 concentration corresponded to an approximate 5.3 % increase in local correlation. We also found higher gestational Σ_5_BDE concentrations were associated with lower global correlation in a single cluster of voxels located within the primary visual cortex. In this cluster, a doubling of gestational Σ_5_BDE concentration corresponded to an approximate 21.5 % reduction in global correlation with other voxels in the brain. Based on our voxelwise findings, we conducted mediation analyses and found that associations between Σ_5_BDE and seven neurobehavioral outcome measures (BASC-BSI, BRIEF-CRI, BRIEF-GEC, CDI Total Score, SCARED Total Score, SCAS Total Score, and SRS Total Score) were significantly mediated by connectivity patterns of the left primary visual cortex (Table 5).

### 4.1. Associations of gestational PBDE concentrations with neurobehavioral outcomes

Previous cohort studies examining PBDE concentrations have reported several links with neurobehavioral outcomes. Briefly, these studies indicate gestational PBDE exposure exerts wide-ranging effects on children’s adaptive, internalizing, externalizing and social behavior problems (Braun et al. 2014, 2017b; Cecil et al., 2024; Chen et al., 2014; Cowell et al., 2015; Eskenazi et al., 2013; Sagiv et al., 2015; Strawn et al., 2022). Gestational PBDE concentrations have also been associated with lower IQ at 5 years (Chen et al., 2014), poorer attention at 5–7 years (Eskenazi et al., 2013; Roze et al., 2009), poorer attention and working memory at 9 and 12 years (Sagiv et al., 2015) and deficits in executive function at 12 years (Cecil et al., 2025). Our results are consistent with these previously published studies, but the associations reported in this manuscript are derived from a subset of some of these previous studies at age 12 (Cecil et al. 2024, 2025; Strawn et al., 2022).

### 4.2. Neural correlates of gestational PBDE concentrations

The effect of gestational PBDE exposure on functional connectivity has not been extensively studied. To our knowledge, only two studies have previously examined gestational PBDE concentrations with rsfMRI (de Water et al., 2019; Margolis et al., 2020), and those studies examined younger children (age 5), had relatively small sample sizes, and used a graph-theoretical approach for their analyses that provide a measure of how well information can be transmitted across the entire network; thus, a direct comparison with the results of this study is not a like-for-like comparison. In the current study of young adolescents in the HOME Study, higher gestational Σ_5_BDE serum concentrations were associated with lower global correlation in a cluster spanning the left lingual gyrus, left intracalcarine cortex, right supracalcarine cortex, and left cuneal cortex – all regions that play a role in visual processing. The rsfMRI study by de Water et al. examined 34 children at age 5 from a longitudinally followed birth cohort and found higher gestational PBDE serum concentrations (derived by weighted quantile sum analysis) were associated with higher global efficiency of brain areas involved in visual attention and learning, specifically, the hippocampus, lingual gyrus, inferior occipital gyrus, superior occipital gyrus, inferior and middle temporal gyri (de Water et al., 2019). Higher global efficiency of brain areas involved in visual attention was also associated with more executive function problems. De Water et al. also identified associations between higher gestational PBDE serum concentrations and lower global efficiency in the middle occipital gyrus, superior temporal gyrus and precentral gyrus, regions involved in visual, auditory and sensorimotor processing, respectively (de Water et al., 2019). Margolis et al. examined the reading network in 33 children at age 5 from a community sample with rsfMRI. They found that higher gestational PBDE serum concentrations were associated with lower global efficiency of the reading network (Margolis et al., 2020). Higher global efficiency of the reading network was associated with better word reading, however, increased gestational PBDE concentrations were not associated with reading ability. In related work from the HOME Study, however without fMRI, Zhang et al. found gestational PBDE concentrations were inversely associated with reading skills and full-scale intelligence quotient in 8-year-old children (Zhang et al., 2017). Together, these studies indicate that gestational PBDE exposure may alter the architecture associated with the reading network (i.e., at age 5) which may impact later reading abilities (i.e., at age 8).

Notably, our results indicate that gestational BDE-153 exerts a different connectivity association than do the other congeners. During early adolescence, higher gestational BDE-153 serum concentrations were associated with stronger local correlation in the left inferior lateral occipital cortex, a visual area involved in object recognition (Grill-Spector et al., 1998a; Grill-Spector et al., 1998b; Grill-Spector et al. 2000; Lerner et al., 2008; Lerner et al., 2001; Malach et al., 1995) and face perception (Nagy et al., 2012).

Another report from the HOME Study found a 10-fold increase in gestational serum PBDE-153 was associated with poorer behavior regulation at ages 5 and 8 years using the caregiver reported BRIEF (Vuong et al., 2016). Vuong et al. employed a multiple informant model to evaluate *childhood* PBDE concentrations during several exposure windows (1, 2, 3, 5, and 8 years) for participants of the HOME Study (Vuong et al., 2018c). Significant adverse associations were also reported between a 10-fold increase in concentrations of BDE-28 and BDE-153 at age 8 years with simultaneously measured behavioral regulation, poorer emotional and impulse control using the BRIEF-2.

BDE-153 may elicit different biological responses after exposure due to its chemical properties that differ from the other congeners. BDE-153 has a half-life of 7 years (Trudel et al., 2011), high octanol-water coefficient partition, and low water solubility, suggesting greater bioaccumulation in lipid-rich regions (Braekevelt et al., 2003; National Toxicology, 2016; Staskal et al., 2006; Tittlemier et al., 2002). BDE-28, −47, −99, and −100 are bioactivated by cytochrome P450 enzymes, producing active hydroxylated metabolites (Erratico et al. 2012, 2013; Feo et al., 2013). However, BDE-153 is not metabolized as readily in the liver, suggesting it is resistant to enzymatic degradation, thus allowing for greater bioaccumulation (Lupton et al., 2009; Qiu et al., 2007). While the underlying mechanisms for how PBDEs elicit developmental neurotoxicity are complex, some evidence indicates that BDE-47, BDE-99, and BDE-153 congeners increase phosphorylation of ERK1/2, or the MAPK pathway, that is implicated in development of the nervous system (Fan et al., 2010). Disruption of this pathway during critical periods of brain development could interfere with neuronal differentiation, synaptic plasticity, and circuit formation, potentially contributing to long-term impairments in executive function, emotional regulation, and behavior, domains frequently affected in youth gestationally exposed to PBDEs.

### 4.3. Multivariate mediation

To our knowledge, this is the first study to show that altered functional connectivity patterns mediate the association between gestational PBDE exposure and several neurobehavioral outcomes in adolescence. Our multivariate mediation models used seed-to-voxel connectivity maps derived from the significant GCOR cluster (see Fig. 2A) to investigate whether patterns of connectivity related to the neural correlates of PBDE exposure may be mediating the associations observed between the serum PBDE concentrations in pregnant mothers and neurobehavioral assessment scores of adolescents at the age 12 study visit.

For each multivariate mediation model, we found that the first 5 (of 15) PDMs accounted for >95 % of the indirect effect with the majority explained in the first 2 PDMs. For each PDM, we estimated path *a* (exposure to connectivity), path *b* (connectivity to neurobehavioral outcome controlling for exposure), and the indirect effect (*ab*). A positive path *a* indicates that higher gestational PBDE concentration leads to relatively more positive connectivity between the seed region and voxels with positive weights and relatively more negative connectivity between the seed region and voxels with negative weights. A positive path *b* indicates that connectivity between the seed region and positive/negative weighted voxels contribute positively/negatively to the neurobehavioral outcome after controlling for PBDE concentration. Conversely, a negative path *b* indicates that connectivity with positive-weighted voxels is associated with poorer outcomes, and connectivity with negative-weighted voxels is associated with better outcomes, after controlling for PBDE exposure.

Our multivariate mediation models revealed that variance in distinct patterns of functional connectivity to the left primary visual cortex can partially explain the associations of gestational Σ_5_BDE concentrations and several neurobehavioral outcomes at the age 12 study visit. For each of these models, we observed PDMs with either positive or negative effects on the outcome variable (see mediation path *b*, Table 5), which we interpret as maladaptive and neurocompensatory responses to Σ_5_BDE concentration, respectively. The significant weights of these PDMs comprise connectivity patterns that span numerous anatomical regions throughout the brain with regions in the left occipital lobe being relatively more represented. Importantly, the connectivity patterns overlap with multiple functional networks including the default mode network, dorsal attention network, executive control network, and the cerebellum. The identification of distributed connectivity patterns, as opposed to a focal effect, is consistent with a breadth of fMRI research that implicates large-scale brain networks and their interactions in neurodevelopmental conditions (Di Martino et al., 2014; Uddin et al., 2024). For instance, atypical function of the salience and default mode networks is a transdiagnostic feature of developmental conditions (Menon, 2023). Alterations in salience network function and connectivity has been linked to social anxiety and social problems, with adolescents with lower levels of these measures exhibiting greater activation in response to changes in the emotional expressions of standardized human faces (Rosen et al., 2018). Consistent with our mediation analysis related to social responsiveness scores, a review of neuroimaging studies of autism spectrum disorder found disrupted visual perception (Chung and Son, 2020). Additionally, other rsfMRI connectivity studies have reported hypoconnectivity between visual and salience processing regions among children and adolescents with autism (Jao Keehn et al., 2021) and hypoconnectivity between visual and default mode networks in toddlers with a subtype of autism (Lombardo et al., 2019).

### 4.4. Strengths and limitations

Our study has several strengths. Foremost is the prospective pregnancy and birth cohort design with longitudinal follow-up as it allowed us to examine gestational serum PBDE concentrations and subsequently obtain rsfMRI data at early adolescence. Second, our voxelwise methodological approach allows for a non-biased and comprehensive characterization of neural correlates of gestational PBDE concentrations. Voxelwise connectivity analyses allow researchers to test hypotheses about brain-wide connectivity effects by creating “maps” of summary measures of connectivity for each voxel. This contrasts with seed-based connectivity analyses, which measure the connectivity between a defined set of voxels given within a selected ROI and another ROI and/or each other voxel in the brain. The seed approach allows researchers to test specific hypotheses about effects localized to the chosen ROI(s). The set of ROIs in such analyses can be expanded to include up to the entire brain but properly controlling Type I error rates quickly reduces statistical power as the number of tests increases. Because the effects of gestational PBDE concentrations on brain function and organization in adolescence are not well characterized, we opted for a voxelwise approach. By computing LCOR and GCOR maps, we were able to estimate the effect of gestational PBDE concentrations on broad measures of global and local intrinsic connectivity.

The HOME Study represents a typically developing cohort with urban, suburban and rural residents from a metropolitan community within the Midwest region of the United States. The HOME Study enrolled pregnant women at 16 weeks’ gestation between 2003 and 2006. For those unable to provide a sample at enrollment, we allowed for sample collection at 26 weeks. For the current study, we decided to maximize the number of participants, but had only 4 participants where we used the 26 weeks’ sample. Given the estimated PBDE half-lives are between two-to-four years, noting BDE-153 at seven years, we regarded the sampling in the second trimester a good representation for gestational exposure of the mother. However, this second semester sample limited the ability to investigate the impact of the PBDE exposure across other trimesters. The gestational serum PBDE concentrations are consistent with those reported from national survey data for the concurrent period (Woodruff et al., 2011). However, enrollment criteria for the original design of the HOME Study required mothers to live in housing constructed prior to 1978, so the findings from these analyses may be less generalizable to other populations. Most of the HOME study participants were non-Hispanic White, with highly educated mothers so the results may not be generalizable to more heterogeneous populations. However, the primary group examined in the analysis was more likely to be non-Hispanic Black and female than those excluded. Non-Hispanic Black pregnant women may be more highly exposed to PBDEs (Varshavsky et al., 2020). Like other birth cohorts, the HOME Study experienced attrition at 12 years. There is a potential for selection bias, as we excluded 238 adolescents with missing data. While the number of participants in this study is relatively large for an fMRI analysis, it remains a modest sample size for an epidemiology investigation and currently features only one imaging assessment timepoint that occurred in early adolescence. Other environmental exposures that correlate with gestational PBDE concentrations and rsfMRI connectivity patterns could contribute to residual confounding. Our prior report from the HOME Study found that concentrations collected at 16 weeks’ gestation of maternal blood lead, maternal serum Σ_6_PCB, maternal serum DDT, maternal serum DDE and a maternal urinary aggregate for total dialkylphosphate organophosphate pesticide metabolites were not significantly associated with gestational PBDE concentrations or adolescent BRIEF-2 T-scores (p-values >0.2) (Cecil et al., 2025). Our prior publications reporting gestational PBDE concentrations with BASC outcomes, SCARED and CDI outcomes, respectively, also found no meaningful change when including maternal blood lead and mercury concentrations in secondary analyses (Cecil et al., 2024; Strawn et al., 2022). Future studies that consider these and other combined exposures are underway and may provide a more comprehensive understanding of the health risks associated with PBDE exposures.

## 5. Conclusions

This study provides evidence that gestational PBDE serum concentrations influence childhood development by altering how regions of the brain responsible for processing visual information interact with other functional networks. While the negative association between gestational Σ_5_BDE serum concentration (that arises from the BDE-28, −47, −99 and −100 congeners) and global correlation differs from the positive association of BDE-153 corresponding with local correlation, these findings are consistent with established neurobehavioral studies of populations with gestational PBDE exposure demonstrating deficits of executive function, behavioral regulation, attention and working memory as well as externalizing and internalizing symptoms. Related patterns of connectivity were shown to have significant positive *and* negative indirect effects on the associations between PBDE concentration and various neurobehavioral outcomes, suggesting that these relationships may be mediated by maladaptive and neurocompensatory responses in brain network dynamics.

## Supplementary Material

Supplementary Material in Word

## Figures and Tables

**Table 1.** Characteristics of HOME Study Participants

Characteristic	BDE-28, Σ_5_BDE (n=143)	BDE-47, BDE-153 (n=171)	BDE-99 (n=170)	BDE-100 (n=169)	Not in analyses (n=238)
**Adolescent sex, female**	84 (58.7%)	99 (57.9%)	99 (58.2%)	98 (58.0%)	121 (50.8%)
**Adolescent age (years, mean, SD)**	12.3 ± 0.7	12.3 ± 0.7	12.3 ± 0.7	12.3 ± 0.7	N/A
**Maternal Primapara**	50 (35%)	61 (35.7%)	61 (35.9%)	60 (35.5%)	115 (48.3%)
**Maternal age at delivery (years, mean, SD)**	29.1 ± 5.8	29.4 ± 5.7	29.4 ± 5.8	29.4 ± 5.8	29.4 ± 5.7
**Pre-pregnancy BMI (kg/m** ^**2**^ **)**	27.0 (6.8)	26.5 (6.7)	26.5 (6.7)	26.5 (6.7)	26.0 (5.8)
**Maternal ethnicity**					
Hispanic	2 (1.4%)	2 (1.2%)	2 (1.2%)	2 (1.2%)	7 (2.9%)
Non-Hispanic	141 (98.6%)	169 (98.8%)	168 (98.8%)	167 (98.8%)	226 (95%)
Did not report					5 (2.1%)
**Maternal race**					
Black	49 (34.3%)	53 (31.0%)	53 (31.2%)	53 (31.4%)	72 (30.3%)
Non-Black	94 (65.7%)	118 (69.0%)	117 (68.8%)	116 (68.6%)	161 (67.6%)
Did not report					5 (2.1%)
**Maternal education at baseline**					
High school or less	36 (25.2%)	39 (22.8%)	39 (22.9%)	39 (23.1%)	58 (24.4%)
Some college/2-year degree	40 (28.0%)	49 (28.7%)	49 (28.8%)	48 (28.4%)	49 (20.6%)
College graduate	45 (31.5%)	53 (31.0%)	53 (31.2%)	53 (31.4%)	66 (27.7%)
Graduate or professional	22 (15.4%)	30 (17.5%)	29 (17.1%)	29 (17.2%)	58 (24.4%)
Did not report					7 (2.9%)
**Maternal education at 12-year visit**					
High school or less	21 (14.7%)	23 (13.5%)	23 (13.5%)	23 (13.6%)	N/A
Some college/2-year degree	47 (32.9%)	57 (33.3%)	57 (33.5%)	56 (33.1%)	N/A
College graduate	42 (29.4%)	49 (28.7%)	49 (28.8%)	49 (29.0%)	N/A
Graduate or professional	33 (23.1%)	42 (24.6%)	41 (24.1%)	41 (24.3%)	N/A
**Household Income at Baseline(K$)**	55 (17.5, 75)	55 (27.5, 85)	55 (27.5, 85)	55 (27.5, 85)	55 (27.5, 85)

Arithmetic mean values and standard deviations are reported for adolescent age and maternal age at delivery. Numbers, and percentages of sub- cohorts are reported for other characteristics. N/A – not applicable

**Table 2.** Gestational serum PBDE concentrations (ng/g lipid) from pregnant women upon study enrollment at 16 weeks or subsequently at 26 weeks gestation between 2003 and 2006.

Analyte	N	Min	Max	Mean	Std	P25	Median	P75	Geomean (95%CI)	Percent above LOD
BDE-28	143	0.3	16.7	1.6	2.0	0.7	1	1.7	1.1 (1 – 1.3)	88.8
BDE-47	171	2.2	379	36.1	52.5	11.6	20.4	38.1	22 (19.1 – 25.2)	99.4
BDE-99	170	0.6	84.6	8.3	11.6	2.6	4.55	8	4.9 (4.3 – 5.7)	99.4
BDE-100	169	0.5	74.1	6.8	10.3	2.2	3.6	7.4	4.0 (3.5 – 4.7)	97.6
BDE-153	171	0.6	152	8.8	15.3	2.4	4.3	8.6	4.9 (4.2 – 5.6)	97.1
∑_5_BDE	143	5.9	561.4	60.1	81	20.7	33.6	70.1	38.1 (32.9 – 44.1)	N/A

Minimum, Min; Maximum, Max; Arithmetic mean, Mean; Standard Deviation, Std; 25^th^ percentile, P25; 75^th^ percentile, P75; Geometric mean, Geomean; 95% confidence interval, 95%CI; Limit of detection, LOD; ∑_5_BDE is calculated as arithmetic sum of BDE-28, −47, −99, −100 and −153 gestational serum concentrations, with LOD not applicable (N/A)

**Table 3:** Pearson correlation coefficients (and p-values) for associations between log_2_BDE serum concentrations from pregnant women and key neurobehavioral assessments for the adolescent at age 12 study visit.

Assessment	Reporter	Measure	BDE-28	BDE-47	BDE-99	BDE-100	BDE-153	Σ_5_BDE
BASC-3	Caregiver	EXT	0.13 (0.117)	0.11 (0.163)	0.08 (0.302)	0.09 (0.227)	***0.19 (0.015)***	0.12 (0.142)
BASC-3	Caregiver	INZ	0.11 (0.186)	0.06 (0.435)	0.06 (0.451)	0.03 (0.703)	0.01 (0.891)	0.08 (0.345)
BASC-3	Caregiver	BSI	***0.19 (0.027)***	0.15 (0.056)	0.12 (0.119)	0.12 (0.133)	***0.17 (0.031)***	***0.18 (0.036)***
BASC-3	Caregiver	AKL	−0.15 (0.067)	−***0.17 (0.029)***	−***0.15 (0.048)***	−0.12 (0.111)	−0.13 (0.098)	−0.12 (0.155)
BASC-3	Adolescent	FII	***0.30 (0.000)***	***0.21 (0.006)***	***0.20 (0.010)***	0.15 (0.052)	0.12 (0.116)	***0.26 (0.002)***
BASC-3	Adolescent	ESI	***0.24 (0.004)***	0.13 (0.089)	0.11 (0.177)	0.06 (0.410)	0.06 (0.428)	0.15 (0.074)
BASC-3	Adolescent	IHI	***0.30 (0.000)***	***0.20 (0.009)***	***0.20 (0.010)***	***0.17 (0.032)***	***0.17 (0.031)***	***0.26 (0.002)***
BASC-3	Adolescent	INZ	***0.29 (0.000)***	***0.21 (0.007)***	***0.19 (0.013)***	0.12 (0.109)	0.09 (0.231)	***0.23 (0.007)***
BASC-3	Adolescent	PAI	−0.14 (0.093)	−0.06 (0.409)	−0.05 (0.553)	−0.02 (0.830)	−0.01 (0.904)	−0.05 (0.524)
BASC-3	Adolescent	SPI	0.08 (0.319)	0.01 (0.901)	0.04 (0.574)	−0.02 (0.779)	−0.04 (0.618)	0.05 (0.523)
BRIEF-2	Caregiver	BRI	***0.19 (0.021)***	0.14 (0.068)	0.13 (0.084)	0.12 (0.119)	***0.21 (0.007)***	***0.18 (0.030)***
BRIEF-2	Caregiver	ERI	0.16 (0.053)	0.12 (0.115)	0.14 (0.060)	0.13 (0.101)	0.12 (0.109)	***0.19 (0.025)***
BRIEF-2	Caregiver	CRI	0.13 (0.129)	0.09 (0.218)	0.07 (0.387)	0.06 (0.440)	0.10 (0.210)	0.09 (0.290)
BRIEF-2	Caregiver	GEC	0.16 (0.054)	0.12 (0.126)	0.10 (0.177)	0.09 (0.230)	0.14 (0.074)	0.14 (0.093)
BRIEF-2	Adolescent	BRI	***0.31 (0.000)***	***0.19 (0.012)***	***0.17 (0.029)***	***0.16 (0.036)***	***0.19 (0.012)***	***0.25 (0.003)***
BRIEF-2	Adolescent	ERI	***0.24 (0.004)***	0.12 (0.105)	0.11 (0.172)	0.08 (0.279)	0.07 (0.345)	***0.17 (0.048)***
BRIEF-2	Adolescent	CRI	***0.33 (0.000)***	***0.16 (0.031)***	0.11 (0.149)	0.13 (0.104)	0.11 (0.168)	***0.20 (0.017)***
BRIEF-2	Adolescent	GEC	***0.32 (0.000)***	***0.17 (0.027)***	0.13 (0.089)	0.13 (0.091)	0.13 (0.099)	***0.22 (0.010)***
SSiS	Adolescent	SS	−0.09 (0.295)	−0.04 (0.612)	−0.06 (0.462)	−0.02 (0.774)	−0.05 (0.530)	−0.06 (0.502)
SSiS	Adolescent	PB	***0.28 (0.001)***	***0.19 (0.014)***	***0.19 (0.012)***	0.12 (0.115)	0.07 (0.396)	***0.21 (0.011)***
SCARED	Adolescent	Total score	***0.30 (0.000)***	***0.26 (0.001)***	***0.28 (0.000)***	***0.18 (0.020)***	0.08 (0.269)	***0.28 (0.001)***
SRS-2	Caregiver	Total score	***0.24 (0.004)***	***0.23 (0.002)***	***0.26 (0.001)***	***0.20 (0.008)***	0.14 (0.061)	***0.28 (0.001)***
SCAS	Adolescent	Total score	***0.33 (0.000)***	***0.25 (0.001)***	***0.24 (0.002)***	0.14 (0.068)	0.05 (0.531)	***0.26 (0.001)***
CDI-2	Adolescent	Total score	***0.26 (0.002)***	***0.16 (0.032)***	***0.17 (0.030)***	0.11 (0.169)	0.06 (0.413)	***0.16 (0.050)***

Key: BASC-3 – Behavior Assessment System for Children, version 3: AKL – Composite Adaptive Skills, BSI – Behavior Symptoms Index, ESI – Composite Emotional Symptoms, EXT – Composite Externalizing Problems, FII – Functional Impairment Index, IHI – Composite Inattention/Hyperactivity, INZ – Composite Internalizing Problems, PAI – Composite Personal Adjustment, SPI – Composite School Problems; BRIEF-2 – Behavior Rating Inventory of Executive Functioning, version 2: BRI – Behavior Regulation Index, CRI – Cognitive Regulation Index, ERI- Emotion Regulation Index, GEC – Global Executive Composite; SSiS – Social Skills Improvement System, SS – Social Skills, PB – Problem Behaviors; SCARED – Screen for Child Anxiety Related Disorders; SRS – Social Responsiveness Scale, version 2; CDI-2 – Child Depression Inventory, version 2; SCAS – Spence Children’s Anxiety Scale

**Table 4:** Details for the clusters showing significant associations between gestational PBDE concentrations and GCOR. Cluster center coordinates are in Montreal Neurological Institute (MNI) space and correspond to the voxel of peak association. Anatomical overlap is the amount of the given anatomical structure that is covered by the corresponding cluster; values are given for each region in the Harvard-Oxford brain atlas for which the cluster spanned at least 5%.

Congener	N	Center (x,y,z)	Volume (cm^3^)	size p-FDR	Anatomical overlap
∑_5_PBDE	143	−20,−58,−04	4.89	<0.0001	23% of LG l, 10% of ICC l, 10% of SCC r, 9% of CC l
BDE-28	143	−22,−56,−04	1.76	0.16	6% LG l
BDE-47	171	−06,−90,+20	4.24	0.0001	14% ICC l, 14% of LG l, 7% CC l, 5% CC r
BDE-99	170	−10,−74,+08	0.86	0.23	7% of ICC l
BDE-100	169	−06,−90,+20	5.06	<0.0001	17% of ICC l, 13% of CC l, 12% of LG l, 5% CC r

Key: l - left, r - right, LG - Lingual Gyrus, ICC - Intracalcalrine Cortex, SCC - Supracalcarine Cortex, CC - Cuneal Cortex

**Table 5:** Results of the multivariate mediation analyses for the significant associations observed between gestational ∑_5_BDE concentrations and each of seven behavioral outcome measures at age 12. Mediation paths (*a* and *b*) and the indirect effect (*ab*) are given for each principal directions of mediation (PDM). The volume listed for each PDM is the total volume of voxels with significant weights as determined by bootstrap testing. Anatomical distribution is percent of voxels with significant weights for each PDM that falls within the given anatomical structure; structures comprising less than 5% of the significant voxels are not listed.

Outcome	Total Effect (path c)	Total Effect p-value	Mediation Model p-value	Mediation Paths (a, b, ab)	Volume (cm^3^)	Anatomical Distribution
BASC-3 BSI (caregiver reported)	1.387	0.035	0.009	PDM 1: 3.59, 0.78, 2.80	10.24	ICC l (12.9%), OP r (8.8%), sLOC r (8.3%), Cuneal l (7.1%), Precuneus (6.6%), CerebVI l (6.6%), Cuneal r (6.3%), TOFusC l (5.6%)
				PDM 2: 2.29, −0.76, −1.74	8.57	LG l (21.9%), ICC l (16.8%), Cuneal l (9.5%), LG r (8.5%), CerebI r (5.6%), Precuneus (5.6%)
				PDM 3: 1.93, 0.30, 0.57	0.62	Cuneal l (42.5%), TOFusC l (11.7%), sLOC l (8.0%), Precuneus (5.8%), LG l (5.4%), CerebVI l (5.2%)
				PDM 4: 1.44, −0.18, −0.25	3.79	LG l (36.7%), Cuneal l (31.9%)
				PDM 5: 1.68, 0.12, 0.21	4.89	OP l (25.6%), ICC l (14.2%), OP r (10.8%), CerebIV/V r (7.1%), OFusG l (6.1%)
BRIEF-2 CRI (adolescent reported)	1.728	0.014	0.016	PDM 1: 3.62, 0.75, 2.73	8.15	sLOC r (16.7%), Precuneus (11.0%), ICC l (10.1%), Cuneal l (9.1%), CerebVI l (6.8%), Cuneal r (6.5%), TOFusC l (5.7%), SCC l (5.5%)
				PDM 2: 2.30, −0.83, −1.92	5.61	LG l (20.5%), LG r (13.2%), Precuneus (10.9%), OFusG l (9.4%), TOFusC l (7.8%)
				PDM 3: 1.98, 0.30, 0.60	5.93	sLOC l (22.0%), ICC l (21.9%), Precuneus (14.0%), Cuneal l (10.8%), LG l (5.5%), CerebVI l (5.1%)
				PDM 4: 1.47, −0.21, −0.31	4.82	LG l (42.6%), ICC l (18.7%), Cuneal l (18.6%)
				PDM 5: 1.87, 0.15, 0.27	9.86	OP l (24.2%), CerebVI l (12.6%), ICC l (9.6%), CerebIV/V l (8.7%), OFusG r (8.5%), OFusG l (8.0%)
BRIEF-2 GEC (adolescent reported)	1.938	0.007	0.046	PDM 1: 3.60, 0.71, 2.55	6.50	sLOC r (15.8%), Precuneus (9.9%), ICC l (9.7%), Cuneal l (9.5%), Cuneal r (6.7%), SCC l (6.1%), CerebVI l (6.0%)
				PDM 2: 2.36, −0.83, −1.96	5.91	LG r (22.1%), LG l (15.2%), Precuneus (12.3%), OFusG l (9.0%), TOFusC l (6.1%)
				PDM 3: 2.02, 0.29, 0.58	5.36	sLOC l (24.0%), ICC l (21.2%), Precuneus (11.9%), Cuneal l (11.0%), CerebVI l (8.7%)
				PDM 4: 1.40, −0.20, −0.27	3.61	LG l (38.1%), Cuneal l (22.8%), ICC l (19.5%)
				PDM 5: 1.83, 0.14, 0.26	9.79	OP l (24.0%), ICC l (13.1%), OFusG r (9.8%), CerebVI l (8.7%), OFusG l (6.3%), CerebIV/V l (5.6%)
SCARED Total score (adolescent reported)	3.575	<0.001	0.007	PDM 1: 3.57, 0.98, 3.51	12.32	Precuneous (11.0%), LG l (7.7%), TOFusC l (6.9%), iLOC l (6.6%), Cereb6 l (6.6%), Cuneal l (6.5%), OFusG l (6.2%), ICC l (5.8%), Cereb45 l (5.5%), Cuneal r (5.2%)
				PDM 2: 2.44, −1.08, −2.63	10.70	OP r (11.6%), ICC r (11.4%), sLOC l (8.9%), ICC l (7.1%), LG r (6.6%), Precuneous (6.1%)
				PDM 3: 2.27, 0.29, 0.65	12.40	OFusG l (15.2%), OP r (10.5%), OP l (8.7%), TOFusC r (8.3%), Cuneal l (7.9%), OFusG r (7.2%), sLOC r (6.2%), LG l (5.6%), sLOC l (5.3%)
				PDM 4: 1.56, −0.23, −0.36	2.73	sLOC r (10.0%), Cuneal l (9.9%), LG r (9.6%), MidFG l (8.3%), OP r (7.0%), OP l (6.0%), IFG tri l (5.6%), SFG l (5.2%), aITG r (5.1%)
				PDM 5: 1.41, 0.23, 0.32	17.12	LG l (18.0%), ICC l (10.6%), OP l (9.2%), iLOC r (7.2%), iLOC l (6.8%), TOFusC l (6.6%), OFusG l (6.6%), Cereb6 l (5.8%), Cuneal l (5.6%)
SRS-2 Total score (caregiver reported)	1.996	0.001	0.001	PDM 1: 3.52, 0.76, 2.66	8.74	Precuneus (12.4%), ICC l (12.0%), TOFusC l (11.8%), CerebVI l (10.4%), Cuneal l (6.5%), Cuneal r (5.9%), SCC l (5.9%)
				PDM 2: 2.43, −0.88, −2.15	2.30	LG l (31.6%), LG r (11.8%), ICC l (10.9%), TOFusC l (10.1%), CerebVI l (8.0%), OFusG l (7.0%)
				PDM 3: 1.94, 0.29, 0.57	6.62	ICC l (24.5%), sLOC l (14.3%), LG l (9.5%), Precuneus (8.9%), TOFusC l (7.9%), TOFusC r (7.3%), CerebVI l (6.5%)
				PDM 4: 1.47, −0.20, −0.29	6.22	LG l (30.5%), Cuneal l (26.8%), ICC l (9.2%), Cuneal r (7.1%)
				PDM 5: 1.76, 0.14, 0.25	7.57	OP l (14.9%), OP r (9.9%), ICC l (9.8%), OFusG r (7.8%), CerebVI l (7.2%), CerebIV/V r (6.9%)
SCAS Total score (adolescent reported)	2.278	0.002	0.008	PDM 1: 3.51, 0.88, 3.10	4.78	Precuneus (12.7%), CerebVI l (10.8%), TOFusC l (8.3%), ICC l (7.6%), LG l (7.6%), OFusG l (7.5%), iLOC l (6.2%), Cuneal l (5.4%), PostCG l (5.1%)
				PDM 2: 2.43, −0.83, −2.01	3.19	LG r (15.1%), Precuneus (13.6%), Cuneal r (9.7%), OP r (8.8%), ICC l (7.0%), CerebVI l (5.9%)
				PDM 3: 2.17, 0.22, 0.48	5.78	OP l (17.2%), Cuneal l (10.6%), LG l (8.4%), TOFusC r (8.4%), Precuneus (8.2%), OFusG l (6.8%), sLOC l (6.7%), CerebVI l (6.5%)
				PDM 4: 1.54, −0.19, −0.29	4.72	LG l (36.8%), Cuneal l (20.7%), ICC l (19.2%)
				PDM 5: 1.69, 0.14, 0.23	14.44	OP l (19.6%), CerebVI l (9.4%), OFusG r (8.5%), OP r (7.7%), ICC l (6.7%), CerebIV/V l (5.9%), OFusG l (5.3%)
CDI-II Total score (adolescent reported)	1.612	0.023	0.002	PDM 1: 3.55, 0.82, 2.90	7.09	Precuneus (21.7%), Cuneal l (10.2%), ICC l (8.7%), Cuneal r (8.3%), SCC l (5.9%), iLOC l (5.2%), CerebVI l (5.0%)
				PDM 2: 2.44, −0.89, −2.17	0.69	Precuneus (27.9%), TOFusC l (16.3%), LG l (15.6%), MidFG r (10.5%), OFusG l (9.4%)
				PDM 3: 1.98, 0.29, 0.58	7.90	sLOC l (18.8%), ICC l (14.0%), LG l (10.2%), TOFusC r (9.8%), Precuneus (8.3%), Cuneal l (7.0%), OP l (5.5%)
				PDM 4: 1.62, −0.26, −0.42	7.40	LG l (31.9%), ICC l (24.2%), Cuneal l (14.7%), SCC r (6.5%)
				PDM 5: 1.53, 0.18, 0.27	16.15	OP l (17.2%), OFusG r (11.3%), OP r (10.0%), CerebVI l (8.0%), ICC l (6.7%), LG l (6.2%), OFusG l (5.3%)

Key: BASC-3 – Behavior Assessment System for Children, version 3: BSI – Behavior Symptoms Index; SCAS – Spence Children’s Anxiety Scale, CDI – Child Depression Inventory; SRS – Social Responsiveness Scale; BRIEF-2 – Behavior Rating Inventory of Executive Functioning, version 2: CRI – Cognitive Regulation Index, GEC – Global Executive Composite, l - left, r - right, LG - Lingual Gyrus, ICC - Intracalcalrine Cortex, Cereb – cerebellar lobule, OP – occipital pole, sLOC – superior lateral occipital cortex, TOFusC – temporal-occipital fusiform cortex, OFusG – occipital fusiform gyrus, SCC - Supracalcarine Cortex, CC - Cuneal Cortex

## Data Availability

The datasets generated during and/or analyzed for the current study are not publicly available but are available from the corresponding author upon reasonable request. The HOME Study principal investigators have actively engaged in collaborative data-sharing projects. We welcome new collaborations with other investigators. Investigators interested in HOME Study data can explore options at the following location: https://homestudy.research.cchmc.org/and use the available link to contact the investigators to discuss collaborative opportunities. The Data Sharing Committee meets regularly to review proposed research projects and ensure they do not overlap with extant projects and are an efficient use of scarce resources (e.g., biological samples). Funds to support staff efforts in the assembly and distribution of data sets are required.

## Appendix A. Supplementary data

Supplementary data to this article can be found online at https://doi.org/10.1016/j.ijheh.2026.114745.
