# Supplementary material for: Gestational PBDE concentrations and functional connectivity in adolescents: The HOME Study
Source: Int J Hyg Environ Health. Author manuscript; Available in PMC 2026 May 7. (PMC13151986; doi:10.1016/j.ijheh.2026.114745)
Supplement: Supplementary Material in Word [file NIHMS2170015-supplement-Supplementary_Material_in_Word.docx]

**Supplemental Material**

**Gestational PBDE Concentrations and Functional Connectivity in Adolescents: The HOME Study**

Jonathan Dudley, Kimberly Yolton, Alex D. Edmondson, Yingying Xu, Aimin Chen, Jeffrey R. Strawn, Joseph M. Braun, Andreas Sjodin, Bruce P. Lanphear, and Kim M. Cecil


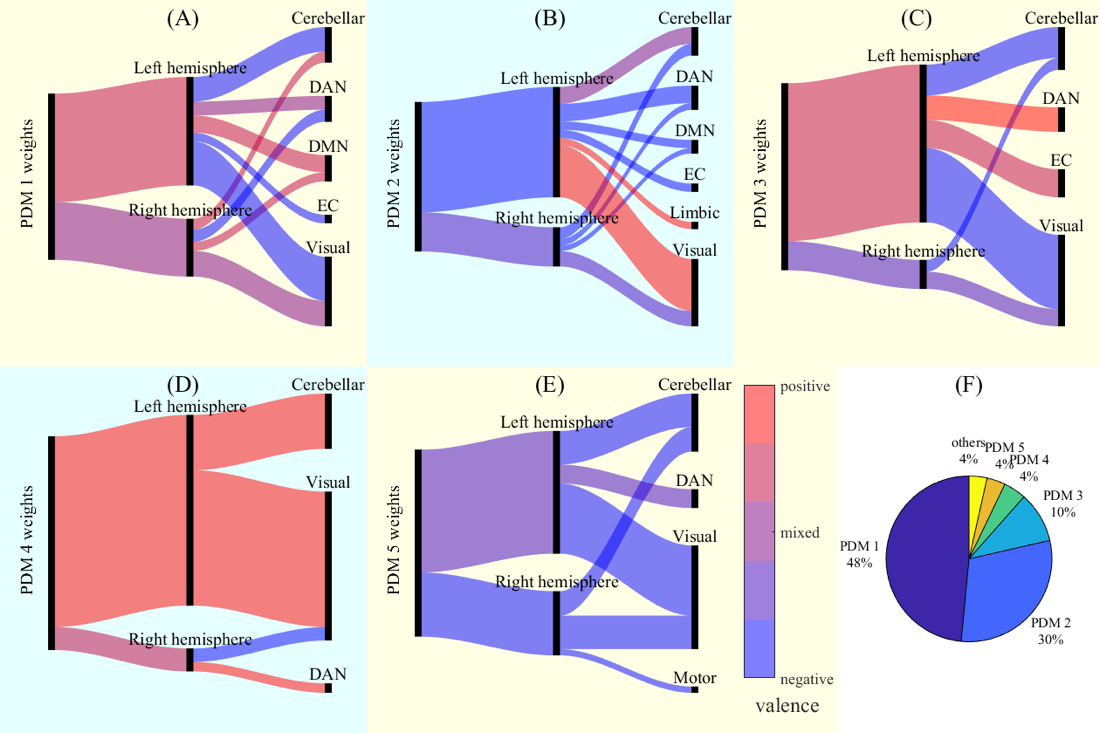


**Supplemental Figure 1**. A multivariate mediation model showing a significant indirect effect on the observed positive association between gestational log_2_Σ_5_BDE concentration and Behavior Assessment System for Children Behavior Symptoms Index (BSI) obtained at the age 12 study visit. Parts A-E show the distribution of significant weights across hemispheres and functional networks for each of the first five PDMs. Heights of the vertical black bars are proportional to the volume of significant weights. Links are color coded from blue to purple to red and indicate the valence of the weights; positive/negative weights indicate that higher levels of gestational ∑_5_BDE exposure are associated with increased/decreased connectivity between the primary visual cluster (**Figure 1A**) and the given region. Yellow backgrounds indicate the PDM has a positive path b and thus explain some of the positive association between gestational ∑_5_BDE concentration and BSI; blue backgrounds indicate the PDM has a negative path b and thus exert an ameliorating effect. Part (F) shows the percentage of the model’s indirect effect explained by each PDM. Functional network abbreviations: DAN – dorsal attention network, DMN – default mode network, EC – executive control network.


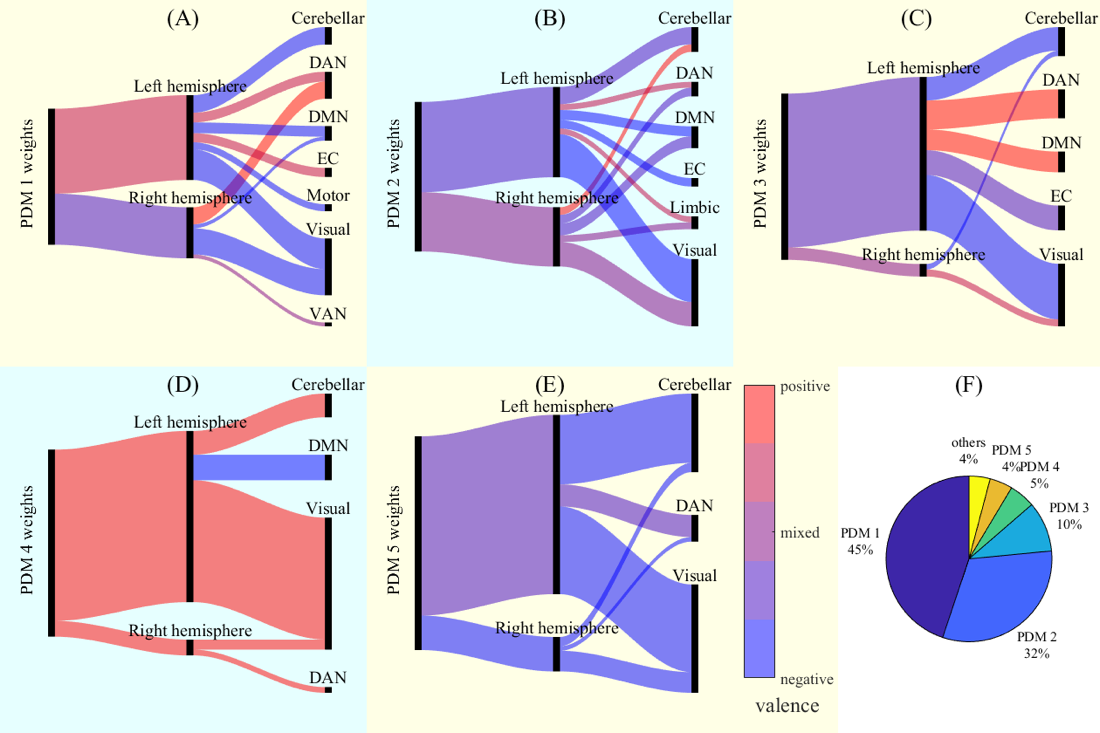


**Supplemental Figure 2**. A multivariate mediation model showing a significant indirect effect on the observed positive association between gestational log_2_∑_5_BDE concentration and Behavior Rating Inventory of Executive Functioning Cognitive Regulation Index (CRI) obtained at the age 12 study visit. Parts A-E show the distribution of significant weights across hemispheres and functional networks for each of the first five PDMs. Heights of the vertical black bars are proportional to the volume of significant weights. Links are color coded from blue to purple to red and indicate the valence of the weights; positive/negative weights indicate that higher levels of gestational ∑_5_BDE exposure are associated with increased/decreased connectivity between the primary visual cluster (**Figure 1A**) and the given region. Yellow backgrounds indicate the PDM has a positive path b and thus explain some of the positive association between gestational ∑_5_BDE concentration and CRI; blue backgrounds indicate the PDM has a negative path b and thus exert an ameliorating effect. Part (F) shows the percentage of the model’s indirect effect explained by each PDM. Functional network abbreviations: DAN – dorsal attention network, DMN – default mode network, EC – executive control network.


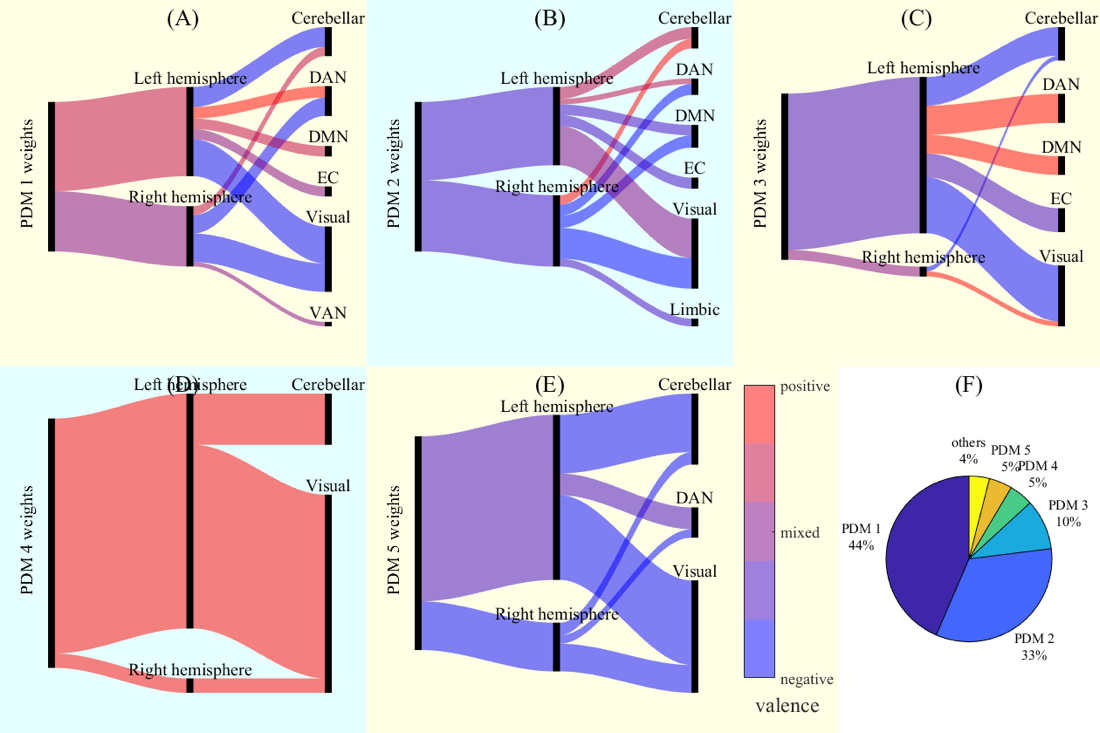


**Supplemental Figure 3**. A multivariate mediation model showing a significant indirect effect on the observed positive association between gestational log_2_∑_5_BDE concentration and Behavior Rating Inventory of Executive Functioning Global Executive Composite (GEC) obtained at the age 12 study visit. Parts A-E show the distribution of significant weights across hemispheres and functional networks for each of the first five PDMs. Heights of the vertical black bars are proportional to the volume of significant weights. Links are color coded from blue to purple to red and indicate the valence of the weights; positive/negative weights indicate that higher levels of gestational ∑_5_BDE exposure are associated with increased/decreased connectivity between the primary visual cluster (**Figure 1A**) and the given region. Yellow backgrounds indicate the PDM has a positive path b and thus explain some of the positive association between gestational ∑_5_BDE concentration and GEC; blue backgrounds indicate the PDM has a negative path b and thus exert an ameliorating effect. Part (F) shows the percentage of the model’s indirect effect explained by each PDM. Functional network abbreviations: DAN – dorsal attention network, DMN – default mode network, EC – executive control network.


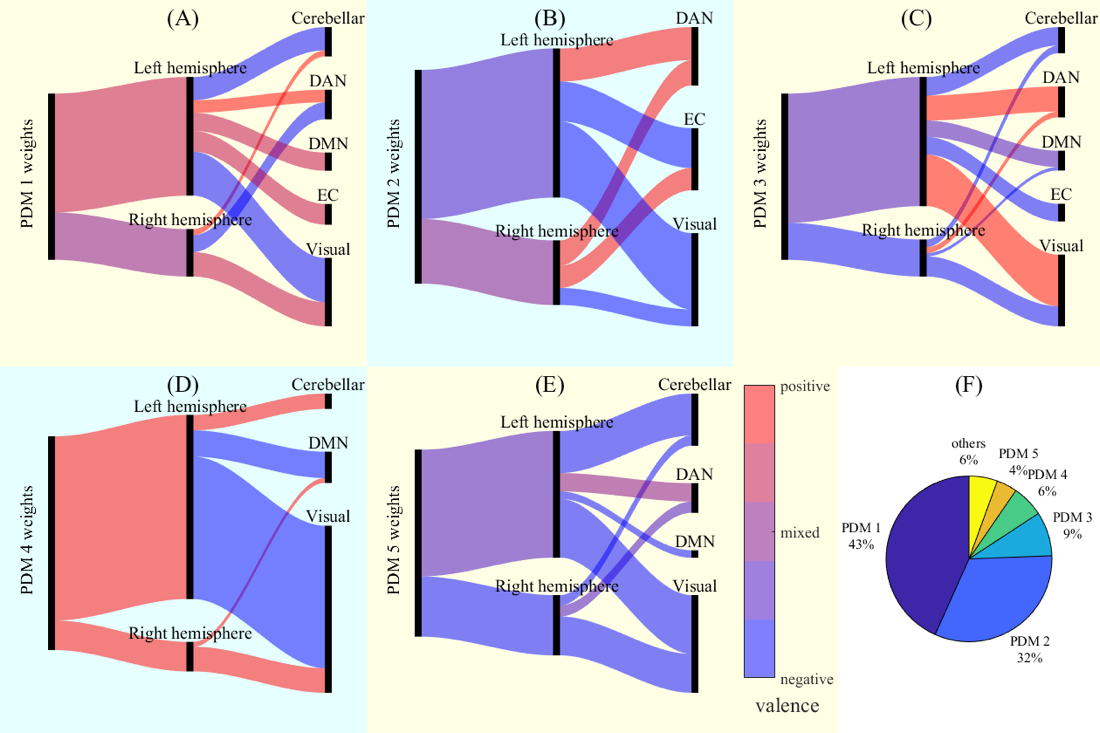


**Supplemental Figure 4.** A multivariate mediation model showing a significant indirect effect on the observed positive association between gestational log_2_∑_5_BDE concentration and Children’s Depression Inventory (CDI) Total T-score obtained at the age 12 study visit. Parts A-E show the distribution of significant weights across hemispheres and functional networks for each of the first five PDMs. Heights of the vertical black bars are proportional to the volume of significant weights. Links are color coded from blue to purple to red and indicate the valence of the weights; positive/negative weights indicate that higher levels of gestational ∑_5_BDE exposure are associated with increased/decreased connectivity between the primary visual cluster (**Figure 1A**) and the given region. Yellow backgrounds indicate the PDM has a positive path b and thus explain some of the positive association between gestational ∑_5_BDE concentration and CDI Total T-score; blue backgrounds indicate the PDM has a negative path b and thus exert an ameliorating effect. Part (F) shows the percentage of the model’s indirect effect explained by each PDM. Functional network abbreviations: DAN – dorsal attention network, DMN – default mode network, EC – executive control network.


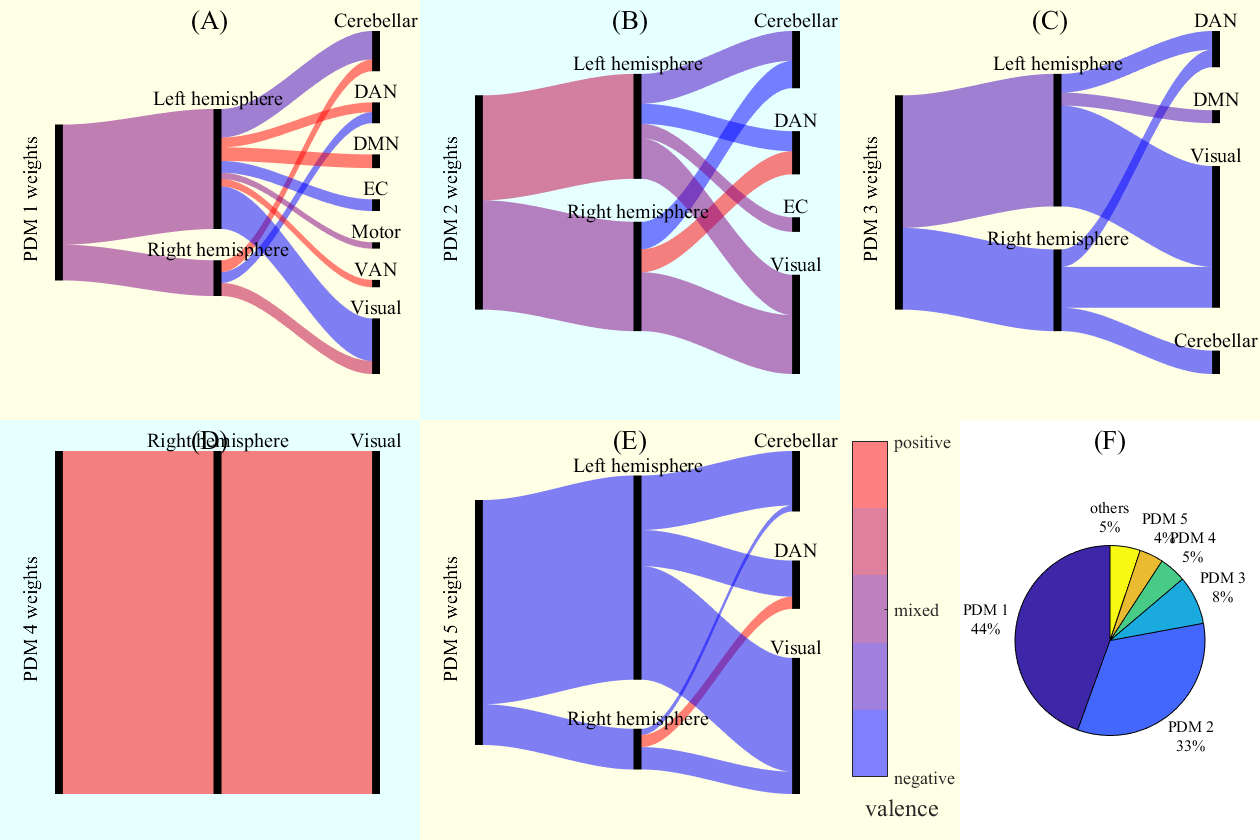


**Supplemental Figure 5.** A multivariate mediation model showing a significant indirect effect on the observed positive association between gestational log_2_∑_5_BDE concentration and Screen for Child Anxiety Related Disorders (SCARED) Total T-score obtained at the age 12 study visit. Parts A-E show the distribution of significant weights across hemispheres and functional networks for each of the first five PDMs. Heights of the vertical black bars are proportional to the volume of significant weights. Links are color coded from blue to purple to red and indicate the valence of the weights; positive/negative weights indicate that higher levels of gestational ∑_5_BDE exposure are associated with increased/decreased connectivity between the primary visual cluster (**Figure 1A**) and the given region. Yellow backgrounds indicate the PDM has a positive path b and thus explain some of the positive association between gestational ∑_5_BDE concentration and SCARED Total T-score; blue backgrounds indicate the PDM has a negative path b and thus exert an ameliorating effect. Part (F) shows the percentage of the model’s indirect effect explained by each PDM. Functional network abbreviations: DAN – dorsal attention network, DMN – default mode network, EC – executive control network.


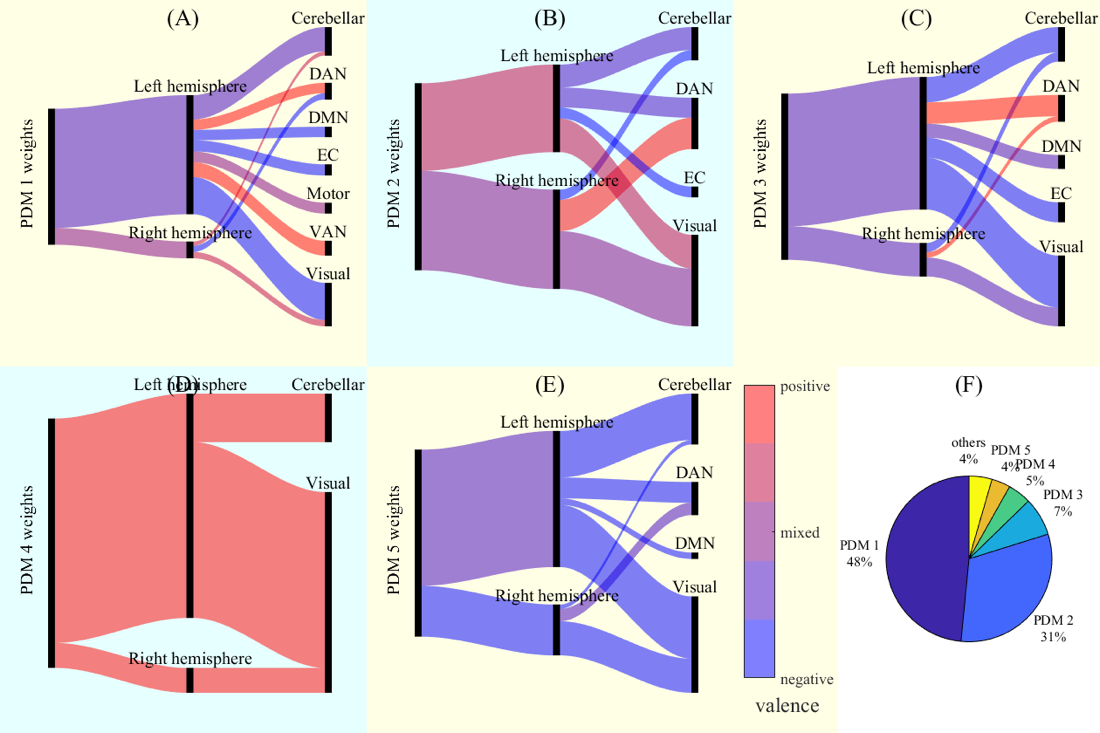


**Supplemental Figure 6**. A multivariate mediation model showing a significant indirect effect on the observed positive association between gestational log_2_∑_5_BDE concentration and Spence Children’s Anxiety Scale (SCAS) Total T-score obtained at the age 12 study visit. Parts A-E show the distribution of significant weights across hemispheres and functional networks for each of the first five PDMs. Heights of the vertical black bars are proportional to the volume of significant weights. Links are color coded from blue to purple to red and indicate the valence of the weights; positive/negative weights indicate that higher levels of gestational ∑_5_BDE exposure are associated with increased/decreased connectivity between the primary visual cluster (**Figure 1A**) and the given region. Yellow backgrounds indicate the PDM has a positive path b and thus explain some of the positive association between gestational ∑_5_BDE concentration and SCAS Total T-score; blue backgrounds indicate the PDM has a negative path b and thus exert an ameliorating effect. Part (F) shows the percentage of the model’s indirect effect explained by each PDM. Functional network abbreviations: DAN – dorsal attention network, DMN – default mode network, EC – executive control network.
